# Effects of exercise on depression and anxiety in university students: a systematic review and meta-analysis

**DOI:** 10.3389/fspor.2026.1708741

**Published:** 2026-05-29

**Authors:** Silje Halvorsen Sveaas, Marthe Brandtzæg, Geir Smedslund, Marte Ørbæk Rognlien, Kjersti Karoline Danielsen

**Affiliations:** 1 Department of Nutrition and Public Health, Faculty of Health and Sport Sciences, University of Agder, Kristiansand, Norway; 2 Unit for HTA Medical Devices, Norwegian Medical Products Agency, Oslo, Norway; 3 Center for Treatment of Rheumatic and Musculoskeletal Diseases (REMEDY), Diakonhjemmet Hospital, Oslo, Norway

**Keywords:** anxiety, depression, exercise, mental health, mental health challenges, physical activity, students in higher education

## Abstract

**Background:**

Although the positive effect of exercise on mental health is well known, its application in the treatment of mental health challenges among students has been limited and lacks systematic evaluation. The aim of this study was to investigate the effect of cardiorespiratory and muscular strength exercise on mental health among students in higher education facing mental health challenges.

**Methods:**

Following PRISMA guidelines, a systematic search was conducted in six databases (Embase, Ovid MEDLINE, and APA PsycInfo via Ovid, CINAHL and SPORTDiscus via EBSCOhost, and Scopus) up to 8 April 2025. The search strategy included terms for the population (university students), intervention (exercise), and outcomes (mental health). Eligible studies included randomized controlled trials or quasi-experimental studies with a control group investigating the effect of cardiorespiratory and muscular strength exercise on mental health outcomes among students in higher education facing mental health challenges. Two independent reviewers performed screening and data extraction. Data were pooled in random-effects models, and standardized mean differences (SMDs) with 95% CI were calculated (SMDs: 0.2–0.4 = small, 0.5–0.7 = medium, and ≥0.8 = large effect sizes). Certainty of the evidence was evaluated according to the Grading of Recommendations Assessment, Development, and Evaluation (GRADE) approach.

**Results:**

Of 6,173 records screened, 11 RCTs and four quasi-experimental trials were included, comprising a total of 659 students. There was low-certainty evidence for large effects of exercise on symptoms of depression [SMD=−1.05 (−1.49, −0.60), *p* < 0.001] and anxiety [SMD=−0.78 (−1.25, −0.31), *p* < 0.01]. There was very low-certainty evidence for the effect of exercise on anxiety sensitivity.

**Conclusion:**

The results suggest medium to large effects of cardiorespiratory and muscular strength exercise on symptoms of depression and anxiety among students in higher education facing mental health challenges. These forms of exercise may be considered as potential treatment modalities for mental health challenges within the student health services.

**Systematic Review Registration:**

PROSPERO CRD42023398623.

## 1 Introduction

An alarming and growing number of students in higher education have reported mental health challenges (1, 2). According to epidemiological studies conducted by the World Health Organization (WHO) across several countries, 20%–35% of students self-reported mental health challenges (3). Similarly, in a recent health survey in Norway, 40% of female and 26% of male students screened positive for mental health challenges, with symptoms of depression, anxiety, and problematic alcohol use being most commonly reported (4). Furthermore, high rates of self-reported self-harm (5) and loneliness (6) among students in higher education have been reported.

The increase in reported mental health challenges might have been partly caused by greater openness in the society regarding mental health challenges and reduced stigma (7). Nevertheless, student life may be a period where individuals are vulnerable to mental health challenges. A recent cohort study involving more than 10,000 young people found that students experienced more mental health challenges than non-students of the same age (8). Student life encompasses several risk factors for mental health challenges such as moving to a new place, academic workload, high levels of perceived stress related to exams, and financial strain (9). Mental health challenges impact the whole person, affecting thoughts, feelings, behavior, and social interactions (10). Consequences of mental health challenges include increased risk of study dropout (11), reduced participation in the workforce (12), and increased risk of chronic physical health conditions (13).

The high prevalence of mental health challenges among students has resulted in a substantial number of students seeking support through student health services (14). The most common treatment modalities for mental health challenges are psychotherapy and antidepressant medications (15–17). Although the mental health benefits of physical activity are well documented (18, 19), and physical activity is recommended in major guidelines for the treatment of various mental health conditions (20, 21), it remains underutilized as a treatment strategy (20, 22).

Physical activity is defined as “any bodily movement produced by skeletal muscles that results in energy expenditure,” while exercise is considered a subset of physical activity that is “planned, structured, and repetitive, and carried out to improve or maintain physical fitness” (23). An umbrella review found that various forms of exercise programs effectively reduced mild to moderate symptoms of depression, anxiety, and psychological distress in both the general population and individuals diagnosed with mental health disorders (18). Furthermore, the effect of exercise on depression is shown to be comparable to the effects of psychotherapy (19). The positive effects of exercise on mental health are attributed to both neurobiological and behavioral mechanisms, including mood enhancement, improved emotion regulation, and increased self-efficacy (24). In addition, qualitative studies highlight that people facing mental health challenges perceive exercise as socially inclusive, providing a sense of meaning and purpose, and contributing to symptom improvement (25). Despite beneficial effects, individuals experiencing mental health challenges may face additional barriers to engaging in exercise, including symptoms of depression and low self-efficacy (26). Moreover, to achieve optimal outcomes, adherence to exercise programs is important as there is a dose–response relationship between physical activity and health (27). Nevertheless, adherence to exercise interventions among people with depression does not appear to be lower than in other clinical populations, and adherence to other treatment strategies such as psychotherapy and antidepressant medication is reported to be equally challenging among people with depression (28).

Among students, existing systematic reviews have generally concluded that exercise interventions have a positive effect on mental health (29–38). However, these reviews often included a wide range of exercise types, with a predominant focus on body–mind exercises such as yoga and tai chi (29–35), or exclusively body–mind exercises (36) or single-session interventions (33). Although any physical activity is better than none, the WHO guidelines emphasize the importance of cardiorespiratory activity at moderate to high intensity and muscle-strengthening activities to promote both mental and physical health (27). Furthermore, most reviews have targeted apparently healthy students in higher education (29, 33–35, 37). Only five reviews have focused on students who are facing mental health challenges (30–32, 36, 38). Nevertheless, these studies include diverse type of interventions (38), focus on only one mental health outcome (30, 31, 36), or are restricted to studies conducted during the COVID-19 pandemic (32). Hence, there is a clear need for a systematic review that specifically investigates the effects of aerobic and muscular strength exercises on mental health among students in higher education facing mental health challenges. This review aimed to address these gaps by focusing on cardiorespiratory and muscular strength exercise, including a broader range of mental health outcomes, and restricting the population to students in higher education facing mental health challenges.

## 2 Methods

A systematic literature review with meta-analysis was conducted to identify and summarize the effects of cardiorespiratory and muscular strength exercise on mental health among university students facing mental health challenges. The protocol was prospectively registered in the PROSPERO register of systematic reviews (CRD42023398623). The review was conducted and reported according to the PRISMA guidelines, ensuring comprehensive reporting and methodological transparency.

### 2.1 Data sources and searches

The search strategy was prepared in collaboration with a university librarian. The complete search strategy is provided in Supplementary File 1. A broad search combining the terms for all types of students in higher education, exercise, and mental health was undertaken. Boolean operators “OR” and “AND” were used to combine synonyms and concept blocks. Synonyms within each concept were combined with “OR” (e.g., university students OR higher education students; exercise OR running OR walking; mental health OR depression OR quality of life). The three concept blocks were combined with “AND” [(e.g., students OR university students OR students in higher education) AND (Exercise OR running OR walking) AND (Mental health OR depression OR quality of life)].

The literature search was conducted across six databases: Embase, Ovid MEDLINE, and APA PsycInfo via Ovid, CINAHL and SPORTDiscus via EBSCOhost, and Scopus. Records were retrived based on terms appearing in titles, abstracts, keywords, or databased-controlled vovabulary where applicable. Searches were initially conducted only through EMBASE, MEDLINE, and APA PsycInfo on 12 October 2023, then updated on 8 April 2025, and adapted to the other databases. Searches were limited to published journal articles.

### 2.2 Eligibility criteria for selection of studies

The research question and eligibility criteria were developed and structured according to the Population, Interventions, Comparators, Outcomes and Study designs (PICOS) framework. Studies published in English as original articles were eligible if they met the following inclusion criteria: (1) The study population had to consist of students in higher education facing mental health challenges. (2) The intervention could be any type of cardiorespiratory or muscular strength exercises (including sports games). Studies in which participants in the exercise group received additional treatments, such as medication or conventional therapy, were only included if the control group received the same supplementary interventions. (3) The control intervention had to be no treatment, a waiting list, or usual/standard care. (4) Mental health had to be an outcome measure (i.e., quality of life, wellness, happiness, psychological distress, or symptoms of depression or anxiety). (5) The study design had to be an RCT or a quasi-experimental study with a control group.

Studies were excluded if they met the following criteria: (1) The intervention consisted of any type of flexibility exercise, dance, taekwondo, or mind–body exercises (e.g., yoga, tai chi, Qigong), a combination of mind–body exercise with cardiorespiratory and/or muscular strength exercise, or a single exercise session. (2) The study population consisted of students with bipolar disorders, psychotic symptoms, attention-deficit/hyperactivity disorders, or sleeping disorders.

### 2.3 Selection of studies

All titles and abstracts retrieved from the database search were screened against the eligibility criteria by at least two review authors independently using the online screening tool Rayyan (39). In addition, all systematic reviews identified in the database search were obtained in full text, and two review authors independently screened the reference lists of these publications. All publications selected through this process were thereafter imported into EndNote V.20 and obtained in full text. All selected full-text publications were assessed by two review authors independently, and disagreements regarding eligibility were resolved through discussion within the reviewer group.

### 2.4 Data extraction

Two review authors extracted results and other relevant information from the included studies. Data on the effects of cardiorespiratory and/or muscular strength exercise on mental health were collected immediately after the intervention period. If both post-intervention and change score were reported, the post-intervention values were extracted. For studies with more than one exercise group, data from these groups were combined into a single exercise group using weighted means based on sample size.

### 2.5 Data synthesis and analysis

Meta-analyses were conducted to summarize the results from original studies when sufficient data were available. Data were pooled in a meta-analysis using Review Manager V5.4.1 software (40). A single, unified dataset extracted by two review authors was entered into the software. For continuous outcome variables, the standardized mean difference (SMD) with 95% CI was calculated. SMDs between 0.2 and 0.4 were considered as small effect sizes, between 0.5 and 0.7 as medium, and ≥0.8 as large (41). A random-effects model was used for all outcomes due to expected heterogeneity among studies. The Cochrane Q test was used to assess heterogeneity, and the I^2^ index was used to estimate the percentage of variability in results across studies attributable to real differences and not chance. Subgroup analyses were based on study design (studies grouped into randomized controlled trials and quasi experimental studies). A *p*-value of <0.05 was considered statistically significant. Results not included in the meta-analysis were summarized in the text.

### 2.6 Quality assessment

Risk of bias for each trial was assessed with the Cochrane Risk of Bias tool, based on published material, by two review authors. Any disagreements were resolved through discussion until agreement was reached. Risk of bias were evaluated for each trial for the following: (1) random sequence generation: low risk if the randomization process was described as truly random; (2) allocation concealment: low risk if a concealed allocation process was described as satisfactory; (3) incomplete outcome data: low risk if the follow-up rate was ≥85%; (4) performance bias: low risk if the participants were blinded, a criteria none of the trials met as the intervention consisted of exercise; (5) blinding of outcome assessments: low risk if outcome measure were blinded, a criterion none of the trials met as the outcome measures were self-reported; (6) selective reporting: low risk if the study reported means with SD for all relevant outcomes and referred to a published protocol in the article; and (7) other bias: low risk if no other source of bias was identified in the trial.

### 2.7 Certainty of evidence

To assess the certainty of the evidence for all outcomes across trials, the Grading of Recommendations Assessment, Development, and Evaluation (GRADE) approach was used. The certainty of evidence was divided into four levels: high, moderate, low, and very low certainty. The following factors were evaluated: risk of bias, inconsistency of results, indirectness of evidence, imprecision, and publication bias (42).

## 3 Results

### 3.1 Study selection

**Figure 1:**
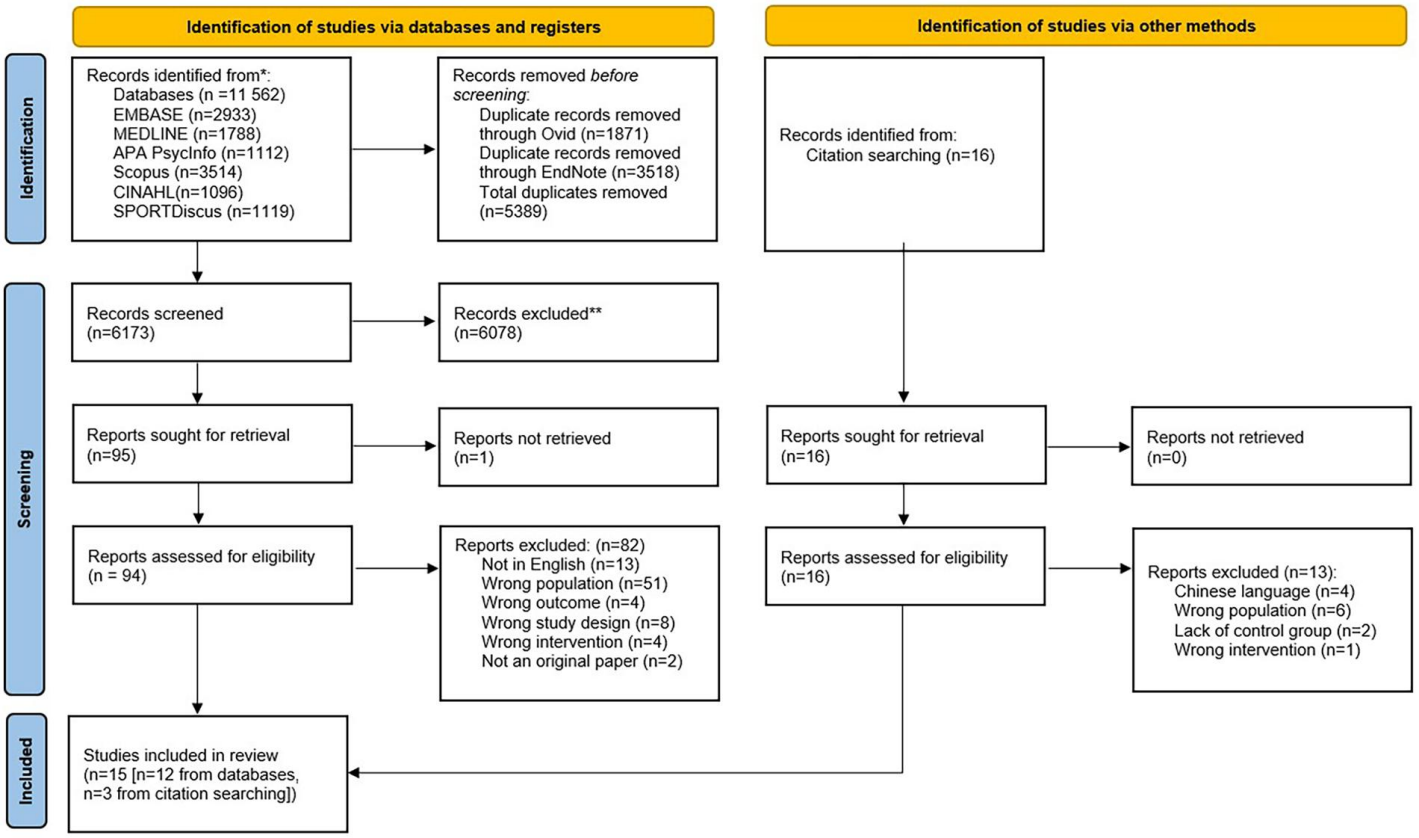
Flowchart of the selection process.

An overview of the selection process of studies is presented in Figure 1. After duplicates were removed, the literature search retrieved 11,562 records. Of these, 95 records were assessed in full text for eligibility, and 12 studies were included. In addition, eight systematic reviews were identified in the literature search. Through citation searching of these reviews, 16 records were assessed for eligibility in full text, and three of these studies were included. Hence, a total of 15 original studies were included in this review. A list of excluded studies with reasons is provided in Supplementary File 2.

### 3.2 Study characteristics

 Table 1 provides a brief overview of the 15 included studies. The studies were published between 1986 and 2023. Five (43–47) of these were conducted in China, five (48–52) in the USA, three (53–55) in Iran, and one in Belgium (56). Eleven (43, 44, 48–50, 52–57) studies were RCTs and four studies (45–47, 51) were quasi-experimental studies with a control group.

**Table 1:** Overview of included studies on the effect of exercise on mental health for students facing mental health challenges.

Study Risk of bias*	Study population *n* (% women), Mean age (SD)	Intervention	Duration	Frequency Length	Outcome measures
Randomized controlled trials					
He 2022 (43) China 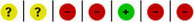	Students with depression *n* = 36 (69%) Mean age not given	EG: Cardiorespiratory exercise: walking, running (medication and psychotherapy) CG: Medication and psychotherapy Adherence: Not reported	3–4 weeks	4–7/week 40–60 min	Depressive symptoms (HAM-D24)
Philippot et al. 2022 (56) Belgium 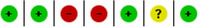	Students with symptoms of anxiety *n* = 30 (83%) Mean age: 21 (2) years	EG: HIIT exercise. Muscular strength and cardiorespiratory exercises in intervals using body weight, 80% of HR_max_. Delivered online CG: No treatment Adherence: Mean participation rate in the EG was 87%	4 weeks	3/week 10 min	Anxiety Depression (DASS-21)
Li et al. 2022 (44) China 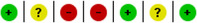	Students with anxiety *n* = 27 (100%) Mean age: 22 years	EG: Muscular strength exercise. 70% of 1 RM CG: Instructed to maintain their usual habits Adherence: Not reported	8 weeks	2/week 40 min	Anxiety (SAS)
Liang et al. 2021 (57) Malaysia	Students with depression *n* = 40 (100%) Age: 18–20 years	EG: Cardiorespiratory exercise. 50–80% of HR_max_ CG: No exercise Adherence: Not reported	18 weeks	2/week 90 min	Depression (BDI and SDS)
Sadeghi et al. 2016 (53) Iran 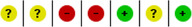	Students with depression *n* = 30 (20%) Mean age: 21 years	EG: Cardiorespiratory exercise, aerobics, HR was monitored manually CG: No treatment Adherence: Not reported	8 weeks	Not given 45–60 min	Depression (BDI)
Medina et al. 2014 (52) USA 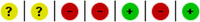	Students with anxiety *n* = 60 (75%) Mean age: 20.7 (5.8) years	EG: Supervised cardiorespiratory exercise. Incline and speed of the treadmill was adjusted to target moderate intensity 70% of HRmax CG: Waitlist control Adherence: Not reported	2 weeks	3/week 20 min	Anxiety sensitivity (ASI)
Hemat-Far et al. 2012 (54) Iran 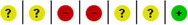	Students with moderate depression *n* = 20 (100%) Age: 18–25 years	EG: Cardiorespiratory exercise. 10 min warm up jogging, 3 sets of running in 6 min at 65–60% HRmax, 3 min relaxing between each running set CG: No treatment Adherence: Not reported	8 weeks	3/week 40–60 min	Depression (BDI)
Atousa 2009 (55) Iran 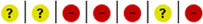	Students with high depression score *n* = 75 (100%) Mean age: not given	EG1: Individual cardiorespiratory exercise (sports) EG2: Cardiorespiratory group exercise, aerobics CG: No exercise Adherence: Not reported	10 weeks	2/week 90 min	Depression (BDI)
Broman-Fulks and Storey 2008 (48) USA 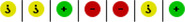	Students with anxiety *n* = 35 (80%) Mean age: 19 (1.6) years	EG: Cardiorespiratory, walking or running on a treadmill at 60–90% of predicted HR_max_ CG: No exercise Adherence: Participants completed no fewer than two and no more than four sessions per week	2 weeks	3/week 20 min	Anxiety sensitivity (ASI-R)
Smits et al. 2008 (49) USA 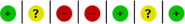	students with anxiety *n* = 39 (75%) Mean age: 21 years	EG: Cardiorespiratory exercise on treadmill. 70% of HR_max_ CG: Waitlist control Adherence: Not reported	2 weeks	3/week 20 min	Anxiety sensitivity (ASI) Anxiety (BAI) Depression (BDI)
Roth and Holmes 1987 (50) USA 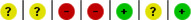	students with anxiety *n* = 36 (51%) Mean age: 19 years	EG: Cardiorespiratory exercise, running or brisk walking. 75% of HR_max_ CG: No treatment Adherence: Not reported	10 weeks	3/week 30 min	Depression (BDI) Anxiety (STAI) Psychological complaints (HSCL-90)
Quasi experimental studies					
Shen 2023 (45) China 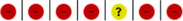	Students with depression *n* = 36 Age and gender not given	EG: Cardiorespiratory exercise, running or basketball CG: No treatment Adherence: Not reported	6 weeks	4/week 45 min	Psychological complaints (HSCL-90)
Zhang et al. 2023 (46) China 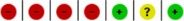	Students with anxiety or depression *n* = 127 Age and gender not given	All groups: cardiorespiratory or muscular strength exercises 60–80% HRmax. Online intervention. EG1: Rope skipping EG2: Aerobic exercise EG3: Muscular strength exercise CG: Did no engage in any physical exercise Adherence: Not reported	3 weeks	3/week 45 min	Depression (SDS) Anxiety (SAS)
Sun et al. 2022 (47) China 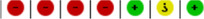	Students with depression *n* = 27 (80%) Age: 17–28 years	EG: Cardiorespiratory, running. 65–75% of HR_max_ CG: No treatment Adherence: Not reported	8 weeks	3/week 25–50 min	Depression (BDI) Pleasure
Williams and Getty 1986 (51) USA 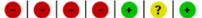	Students with depression *n* = 41 (gender not given) Mean age not given.	EG: Cardiorespiratory exercises. 60–70% of HR_max_ CG: Asked to refrain from aerobic exercise Adherence: Exercise logs turned in every week, subjects who deviated from the protocol were excluded	10 weeks	3/week 50 min	Depression (POMS)

1RM, one repetition maximum; ASI, anxiety sensitivity index, BAI, Beck anxiety inventory; BDI, Beck depression inventory; DASS, depression, anxiety and stress scale; HAMD24, Hamilton depression rating scale; HR_max_, heart rate maximum; POMS, profile of mood states; HSCL, Hopkins symptom checklist; SAS, Zung self-rating anxiety scale; SDS, Zung self-rating depression scale; STAI, state trait anxiety inventory.

*Review authors’ judgment about each risk-of-bias item for each included study (1 = random sequence generation, 2 = allocation concealment, 3 = blinding of participants, 4 = blinding of personnel, 5 = incomplete outcome data, 6 = selective reporting, 7 = other bias).

### 3.3 Participants in the included studies

The 15 studies included a total of 659 participants. The number of included participants ranged from 20 (54) to 127 (46). Most of the included studies comprised both female and male students, although females were overrepresented. Four studies included only female students (44, 54, 55, 57), and three studies did not report gender (45, 46, 51). The mean age ranged from 21 to 25 years, but four studies did not report age (43, 45, 46, 51). Eight studies included students with symptoms of depression (43, 45, 47, 51, 53–55, 57), six studies included students with symptoms of anxiety (44, 48–50, 52, 56), and one study (46) included students with symptoms of anxiety or depression.

### 3.4 Exercise programs in the included studies

The duration of the intervention periods ranged from 2 (48, 49, 52) to 10 weeks (50, 51, 55), and the most common duration was 8 weeks (44, 47, 53, 54). Two interventions were delivered online (46, 56).

Eleven out of the 15 studies involved cardiorespiratory exercise (43, 47–55, 57). One study consisted of either general cardiorespiratory exercise or sports games (45). One study focused solely on muscular strength exercises (44). Another study combined cardiorespiratory and muscular strength exercise (56). One study had three different exercise groups with either rope skipping, aerobic, or resistance exercises (46). In one of the included studies, results from three (46) exercise groups were combined into one group.

Out of the 15 studies, 12 described exercise intensity. Moderate intensity was the most common for cardiorespiratory exercise (47, 49, 50, 52). One study described light intensity (45), two studies reported moderate to light intensity (51, 54), and three studies described light to vigorous intensity (46, 48, 57). Only one study (48) described vigorous intensity. The intervention involving muscular strength exercises specified a load of 70% of one repetition maximum (44).

All studies included reported the duration and frequency of exercise sessions. The most common frequency was three sessions per week (48–50, 52, 54, 56). Session duration varied from 10 min (56) to up to 90 min (55, 57). Adherence to the exercise protocol was only reported by three (48, 51, 56) of the 15 studies, making comparisons of adherence difficult.

### 3.5 Outcome measures in the included trials

#### 3.5.1 Symptoms of depression

Eleven studies reported the effects of exercise on symptoms of depression (43, 46, 47, 49–51, 53–57). All outcome measures for depression were self-reported in questionnaires, and higher scores indicated more severe symptoms. Seven (47, 49, 50, 53–55, 57) studies measured depression using the 21-item Beck Depression Inventory (BDI) with a sum score ranging from 0 to 63 (58, 59). Two studies (46, 57) used the Zung Self-rating Depression Scale (SDS), with 20 items and a sum score ranging from 20 to 80 (60, 61). One study (56) used the Depression Anxiety Stress Scales (DASS) (62), one study (43) used the Hamilton Depression Rating Scale (HAM-D) (63), and one study (51) used the Profile of Mood States (POMS) (64). As the BDI was the most frequently used instrument, data were extracted from BDI in cases where multiple measures of depression were reported. For instance, one study (57) measured depression using both BDI and SDS; in this case, data from BDI were selected for consistency.

#### 3.5.2 Symptoms of anxiety

Five studies examined the effect of exercise on symptoms of anxiety (44, 46, 49, 50, 56). All anxiety outcome measures in the included studies were self-reported through questionnaires, in which higher scores indicated more severe symptoms. Two studies (44, 65) used the Zung Self-rating Anxiety Scale (SAS), comprising 20 questions with a sum score ranging from 0 to 80 (66). One study (50) used the State Trait Anxiety Inventory (STAI), which consists of 20 items and provides a sum score ranging from 20 to 80 (67, 68). One study (56) used DAS21, which includes seven questions for anxiety with a sum score ranging from 0 to 21. Another study (49) used the Beck Anxiety Inventory (BAI), consisting of 21 items and resulting in a sum score from 0 to 63.

#### 3.5.3 Anxiety sensitivity

Three studies examined the effect of exercise on anxiety sensitivity (48, 49, 52), defined as “fear of anxiety-related sensations and their consequences.” All three studies used the Anxiety Sensitivity Index (ASI) as the outcome measure (69). The ASI is a self-reported questionnaire consisting of 16 items, each scored from 0 to 5, in which higher scores indicate greater anxiety sensitivity (69).

#### 3.5.4 Psychological complaints

Two studies reported the effect of exercise on psychological complaints (45, 50), measured using the Hopkins Symptom Checklist (HSCL-90) (70).

#### 3.5.5 Other outcome measures

One of the included studies assessed positive aspects of mental health (47) by measuring pleasure using the Temporal Experience of Pleasure Scale (TEPS) (71). The TEPS is a self-reported questionnaire consisting of 20 items, in which higher scores indicate more pleasure (71).

### 3.6 Risk of bias in included studies

The risk of bias in the included studies is presented in Supplementary File 3. Overall, most studies had a high risk of bias. Three (44, 49, 56) of 11 RCTs had a low risk of selection bias as proper random sequence generation was described, while eight (43, 48, 50, 52–55, 57) RCTs were rated as uncertain for this item due to insufficient information about the randomization process. Only one study (56) described a concealed randomization process.

Blinding of participants and personnel is challenging in exercise studies; hence, only one study (51) had a low risk of performance bias. In addition, since all measures of mental health were self-reported, all included studies had a high risk of detection bias. Overall, a low dropout rate was reported in the included studies. Consequently, only two (48, 55) of the 15 studies had a high risk of incomplete outcome data.

Due to the lack of pre-registered protocols, 12 studies were rated an uncertain for reporting bias, while three studies had a high risk of reporting bias (43, 45, 52). Other sources of bias were low in 12 of the 15 studies. However, three studies had a high risk of other sources of bias due to insufficient description of methodological aspects (43, 55) and no discussion of limitations (45).

### 3.7 Effect of exercise on symptoms of depression

**Figure 2:**
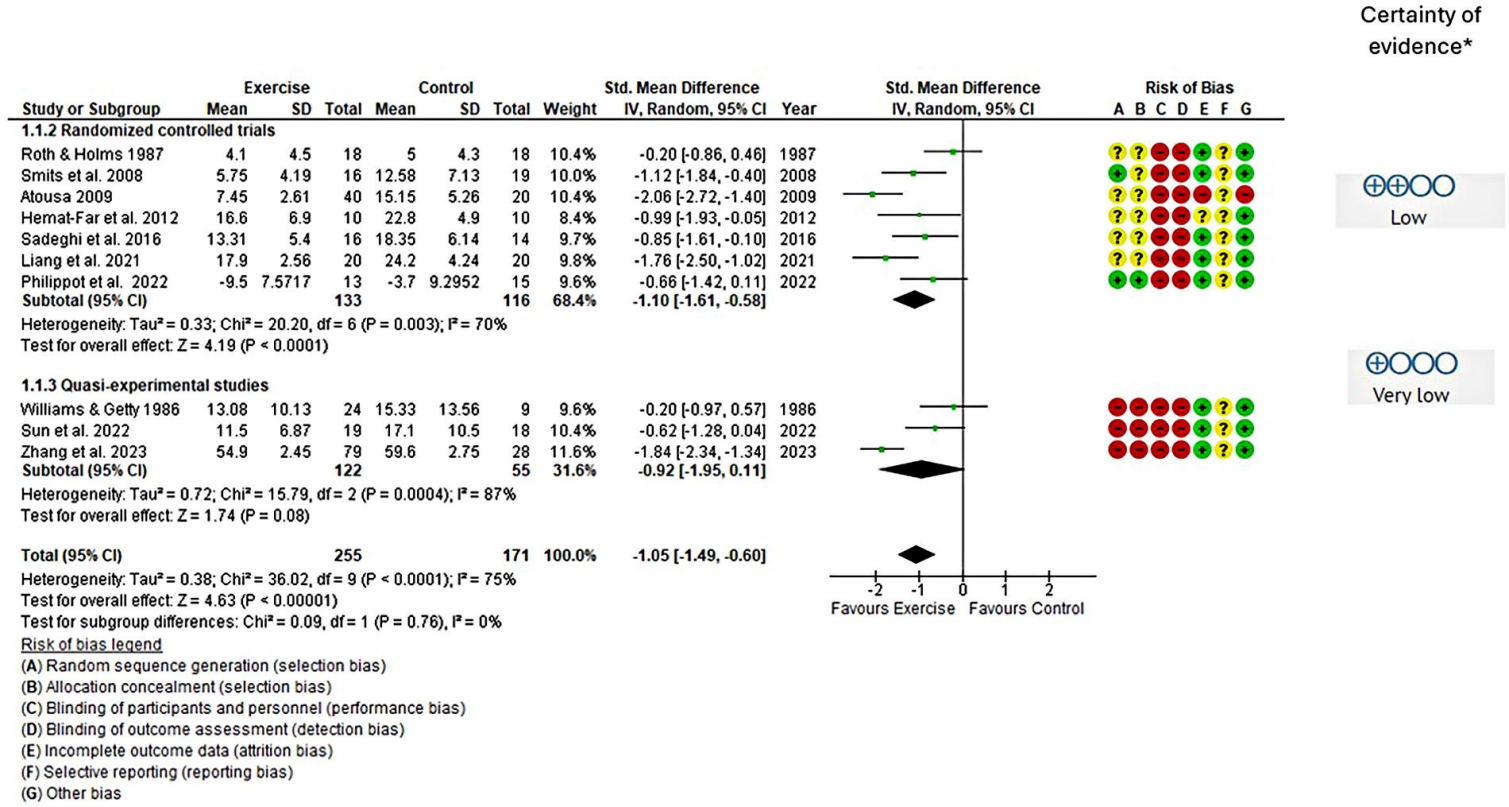
Meta-analyses of effect of exercise on symptoms of depression among students in higher education experiencing mental health challenges. Values are shown as standardized mean difference with 95% CI. * The certainty of the evidence was assessed using the Grading of Recommendations, Assessment, Development, and Evaluation (GRADE) approach.

Ten studies evaluated the effect of exercise on symptoms of depression and provided data for the meta-analysis (46, 47, 49–51, 53–56) (Figure 2). The total result showed a significant large effect of exercise on symptoms of depression among students in higher education experiencing mental health challenges [SMD=−1.05 (95%CI −1.49, −0.60), *p* < 0.001]. Between-study heterogeneity was substantial (I^2^ = 75%).

#### 3.7.1 Randomized controlled trials

Seven RCTs evaluated the effect of exercise on symptoms of depression and provided data to the meta-analysis (49, 50, 53–57) (Figure 2). Results showed low-certainty evidence for a large beneficial effect of exercise on symptoms of depression among students in higher education experiencing mental health challenges [SMD=−1.10 (95%CI−1.61, −0.58), *p* < 0.001]. There was substantial heterogeneity (I^2^ = 70%). Certainty of evidence was downgraded to low due to high risk of bias in the included studies and serious imprecision. One RCT did not provide data for the meta-analysis and reported a significant effect of exercise on symptoms of depression (43).

#### 3.7.2 Quasi-experimental studies

Three quasi-experimental studies evaluated the effect of exercise on symptoms of depression, all included in the meta-analysis (46, 47) (Figure 2). Results showed very low-certainty evidence for no effect of exercise on depression among students in higher education facing mental health challenges [SMD=−0.91 (95%CI −1.95, 0.11), *p* = 0.08]. Between-study heterogeneity was substantial (I^2^ = 87%). Certainty of evidence was downgraded to very low due to serious risk of bias in the included trials and very serious imprecision.

### 3.8 Effect of exercise on symptoms of anxiety

**Figure 3:**
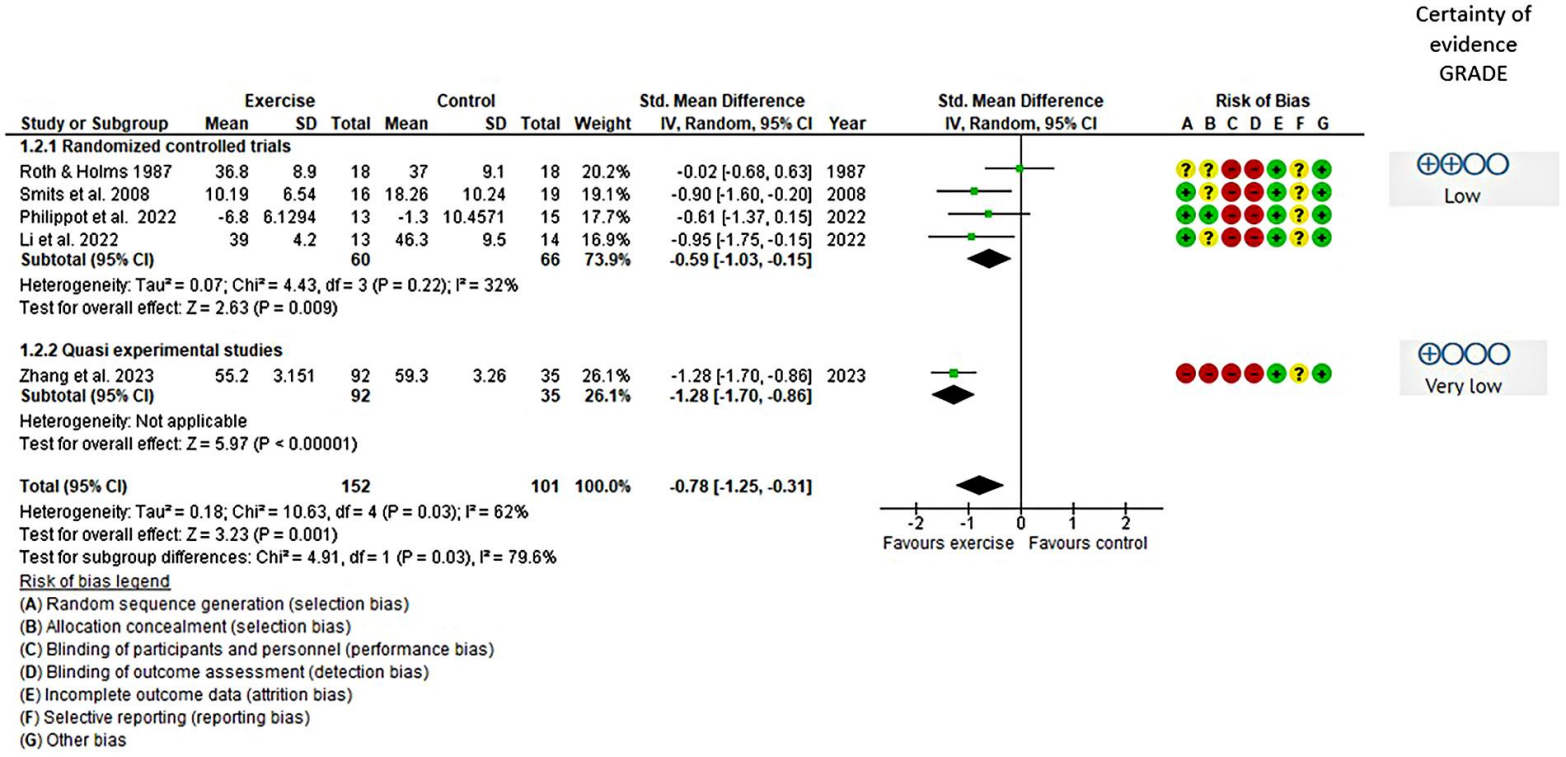
Meta-analyses of the effect of exercise on symptoms of anxiety among students in higher education experiencing mental health challenges. Values are shown as standardized mean difference with 95% CI and separate for RCT and quasi-experimental studies. *The certainty of the evidence was assessed using the Grading of Recommendations Assessment, Development, and Evaluation (GRADE) approach.

Five studies evaluated the effect of exercise on symptoms of anxiety and provided data for the meta-analysis (44, 46, 49, 50, 56) (Figure 3). In total, there was a significant medium to large effect of exercise on symptoms of anxiety among students in higher education experiencing mental health challenges [SMD=−0.78 (−1.25, −0.31), *p* = 0.001]. Between-study heterogeneity was substantial (I^2^ = 62%).

#### 3.8.1 Randomized controlled trials

Four of the included RCTs evaluated the effect of exercise on anxiety and provided data to the meta-analysis (44, 49, 50, 56) (Figure 3). Results showed low-certainty evidence for a medium effect of exercise on anxiety among students in higher education experiencing mental health challenges [SMD=−0.59 (95%CI: −1.03, −0.15), *p* = 0.009]. There was low heterogeneity between the trials (I^2^ ^=^ 32%). Certainty of evidence was downgraded to low due to serious risk of bias in the included trials and serious imprecision.

#### 3.8.2 Quasi-experimental studies

One quasi-experimental study evaluated the effect of exercise on anxiety and reported very low-certainty evidence for a large effect of exercise anxiety [SMD=−1.28 (95%CI −1.70, −0.86), *p* < 0.001] (Figure 3).

### 3.9 Effect of exercise on anxiety sensitivity

**Figure 4:**
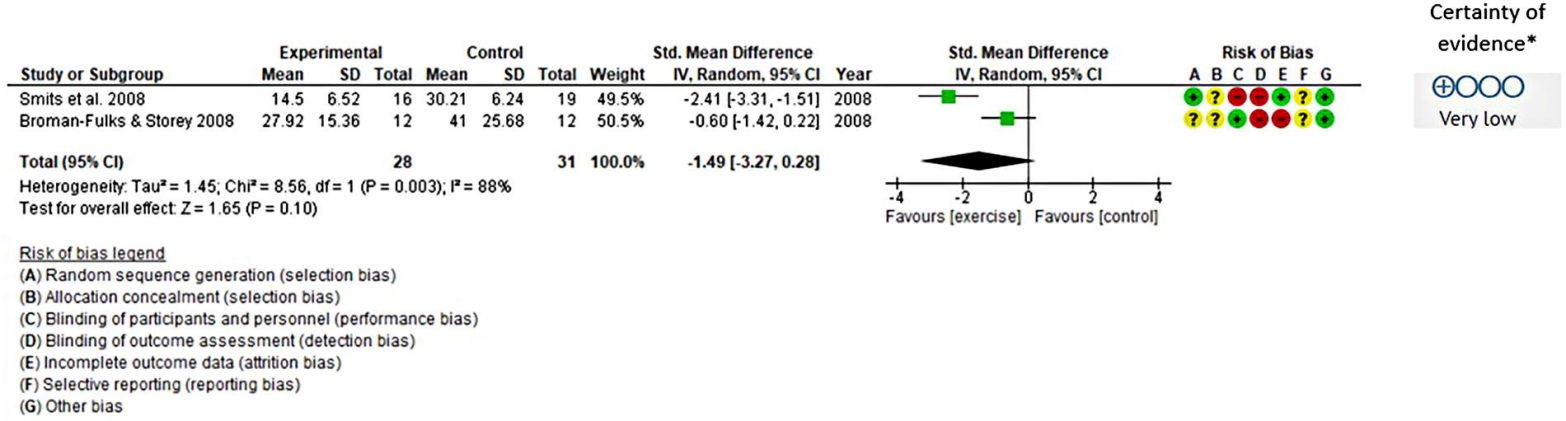
Meta-analyses of the effect of cardiorespiratory and muscular strength exercise on symptoms of anxiety sensitivity among students in higher education experiencing mental health challenges. Values are shown as standardized mean difference with 95% CI. The certainty of evidence was assessed using the Grading of Recommendations Assessment, Development, and Evaluation (GRADE) approach.

Three RCTs evaluated the effect of exercise on anxiety sensitivity (48, 49, 52), with two of these provided data for the meta-analysis (48, 49) (Figure 4). Results showed very low-certainty evidence of no effect of exercise on anxiety sensitivity [SMD=−1.49 (95%CI −3.27, 0.28), *p* = 0.10]. In addition, one study not included in the meta-analysis concluded that exercise had a positive effect on anxiety sensitivity (52). Certainty of the evidence was downgraded to very low due to the small number of RCTs, serious risk of bias in the included studies, and very serious imprecision.

### 3.10 Effect of exercise on other outcomes of mental health

#### 3.10.1 Psychological complaints

One RCT (50) and one quasi-experimental study (45) evaluated the effect of exercise on psychological complaints. The RCT found no effect of exercise on psychological complaints, with the exercise group showing similar scores to the control group on the HSCL at post-test [mean score 0.5 (SD 0.5) vs. 0.5 (SD 0.4)] (50). In contrast, the quasi-experimental study found a significant decrease in psychological complaints following the exercise intervention. After the intervention, the average HSCL-90 score was 172 in the exercise group compared to 208 in the control group (standard deviations were not reported) (45).

#### 3.10.2 Pleasure

One quasi-experimental study (47) evaluated the effect of exercise on pleasure and reported that the running group reported significantly higher pleasure scores than the control group (*p* = 0.049).

## 4 Discussion

The aim of this systematic review was to summarize the effect of cardiorespiratory and muscular strength exercise used as a treatment modality among students in higher education facing mental health challenges. A total of 15 studies, including 659 students, were eligible. The results showed low-certainty evidence for medium to large effects of cardiorespiratory and muscular strength exercise on symptoms of depression and anxiety. There was very low-certainty evidence for effects of these types of exercise on anxiety sensitivity. The interventions consisted of cardiorespiratory exercise.

Our findings are consistent with previous systematic reviews reporting small to medium effects of cardiorespiratory exercise on symptoms of depression and anxiety among students in higher education diagnosed with depression (38) and anxiety (30). However, the effect sizes in the present review, an SMD of −1.05 for depression and −0.78 for anxiety, were generally larger than those reported in earlier reviews (30, 38). Another review of interventions among depressed students included not only cardiorespiratory exercise but also meditation and traditional Chinese exercise (30). Within this review, 17 original studies on cardiorespiratory exercise were analyzed, and an SMD of −0.5 was reported for both depression and anxiety symptoms (38). Despite difference in effect sizes, the consistency in findings across these reviews supports the validity of our results.

We found beneficial effects on mental health from exercise programs that mainly consisted of cardiorespiratory exercise. However, a broad range of exercise modalities has shown moderate effects on symptoms of depression and anxiety among students (30–32, 34–37). This may be explained by the fact that exercise has the potential to improve mental health through multiple mechanisms. The effects of exercise on mental health are understood to arise from a synergy between neurobiological processes and behavioral learning mechanisms, whereby exercise induces acute feelings of pleasure, enhances self-efficacy, and strengthens self-regulatory skills (24). Furthermore, qualitative research has found that exercise interventions foster experiences of social belonging, identity, and meaning among adults facing mental health challenges (25). Nevertheless, the choice of exercise modality influences the outcome of exercise, as it influences physiological processes and physical fitness in distinct ways (72).

In addition to mode of exercise, the optimal dose (frequency, duration, and intensity) for reducing symptoms of depression and anxiety remains controversial. In the present review, only three of the included studies reported adherence to exercise, meaning the actual dose of exercise remains unknown. Most of the interventions were of moderate intensity, with only one study (56) describing high-intensity exercise, which limited the ability to assess differences in effect across varying intensity levels. On the one hand, given that both the intensity and overall dose of exercise were generally low in the included studies, it is plausible that exercise programs incorporating higher intensities and greater exercise volumes may have the potential for greater effect on mental health. On the other hand, despite the low dose of exercise, moderate to large effects were observed on symptoms of depression and anxiety, indicating that small amount of exercise can make a difference for mental health. Another review reported that regardless of mode, light, moderate, and vigorous exercises were equally effective in reducing symptoms of depression, anxiety, and stress among apparently healthy students in higher education (37). In contrast, in various adult populations, higher exercise intensities are associated with greater improvements in symptoms of depression and anxiety (18, 19). Further research is warranted to determine the optimal dose of exercise interventions for students in higher education facing mental health challenges.

Although the present review found a significant effect of exercise on symptoms of anxiety, the effect on anxiety sensitivity was uncertain. This conclusion was based on findings from only two small RCTs (48, 49). One of these trials reported a beneficial effect of exercise on anxiety sensitivity (49), while the other found no significant difference between the exercise and the control groups, despite observing a reduction in anxiety sensitivity within the exercise group following the intervention (48). Given that exercise elicits many of the physiological responses associated with anxiety, but typically without the accompanying discomfort, it has been hypothesized that repeated exposure to these sensations through exercise may lead to a reduction in anxiety sensitivity over time (73). Hence, future exercise studies should include anxiety sensitivity as an outcome measure.

## 5 Practical application

Given the large number of students in higher education reporting mental health challenges (1, 2), there is a need for easily accessible strategies that students themselves can use to strengthen their mental health. Exercise shows potential in this respect, representing a cost-effective and accessible intervention that may also help reduce the burden on the healthcare system. Notably, the effects of exercise on symptoms of depression have been shown to be comparable to those of psychotherapy (19). An advantage with cardiorespiratory and muscular strength exercise is the beneficial impact on physical health (72), offering positive health side effects that other treatment modalities do not provide. Importantly, individuals experiencing mental health challenges are at an increased risk for developing physical diseases, particularly cardiometabolic diseases (74). Hence, the finding of a positive treatment effect of cardiorespiratory and muscular strength exercise on symptoms of depression and anxiety among students in higher education highlights its potential as a valuable intervention and underscores its importance in the management of mental health challenges within student health services.

When integrating exercise into mental health management, the delivery format of exercise should be emphasized. There was a substantial variation in how the exercise interventions were delivered across the included studies. In two of the included studies, the exercise intervention was delivered online (46, 56), and these showed effects on symptoms of depression and anxiety comparable to the other studies (56). However, supervision by exercise professionals, face-to-face contact, and social support during exercise are factors known to enhance exercise adherence among individuals facing mental health challenges (28), and these elements are more difficult to achieve in an online format. In addition, some of the effects of exercise may be explained by social belonging (25). Given that more than half of students report experiencing loneliness (6), in-person exercise session may offer particular advantages. In the present review, few of the studies described group-based exercise interventions. Nevertheless, as supportive social networks are reported to promote mental health among students (9), students should be encouraged to exercise together with others. The use of group exercise is also supported by a comprehensive systematic review of 218 studies, which reported a stronger effect of group exercise compared with individual exercise when used as treatment for depression (19). Hence, the effectiveness of supervised, group-based exercise programs delivered within student health services should be further investigated.

## 6 Future research

There is a need for high-quality RCTs to investigate the effects of cardiorespiratory and muscle strength exercise among students in higher education. Further research is needed to determine the optimal modality, dose, and delivery of exercise for this population, as well as explore how exercise interventions can be effectively implemented within student health services. Future studies should report adherence to exercise and include positive mental health outcomes, such as quality of life, wellbeing, self-efficacy, pleasure, and enjoyment. Moreover, exercise interventions should follow established guidelines regarding the quantity and quality of exercise required to develop and maintain cardiorespiratory fitness and muscular strength.

## 7 Strengths and limitations

Strengths of this systematic review include the comprehensive literature search across six databases and citation tracking of reference lists from existing systematic reviews within this field, which increased the likelihood of identifying all relevant studies. However, a potential limitation is that the search did not include additional databases such as Web of Science. Nevertheless, it has been reported that Scopus, one of the databases we included, covers 99% of the content in Web of Sciences (75). The screening process and data extraction were conducted independently by two reviewers. Additional strengths include the use of meta-analyses and the application of the GRADE framework to assess certainty of the evidence. However, limitations include the restriction to English-language studies. Several studies were excluded due to being published in Chinese, and it remains unknown whether inclusion of these studies would have influenced the results. The inclusion of quasi-experimental studies without randomization may have also introduced selection bias. To address this, results for all outcomes were presented separately for RCTs and quasi-experimental studies. Furthermore, lack of subgroup analyses related to different exercise modalities, intensities, frequencies, durations, and adherence to exercise is another limitation of the present review. However, there were insufficient studies within specific categories to conduct meaningful subgroup analyses. For example, only one study examined a muscle-strengthening program, only one study examined high-intensity exercise, and only three studies reported exercise adherence.

In systematic reviews, the degree to which one can trust the evidence depends on the quality of the studies included. In the present review, the overall certainty of evidence was rated as low or very low across all outcomes due to high risk of bias in the included studies. This bias was largely related to the lack of blinding of participants and outcome measures. Blinding of participants is challenging in exercise interventions, as participants are aware of whether they are engaging in exercise or not. Similarly, blinding outcome measures related to mental health is difficult, given that these outcomes are typically self-reported. Consequently, the assessment of risk of bias may have been overly stringent. The Cochrane Risk of Bias tool, which was used in this review, is primarily designed for pharmacological studies with objectively measured outcomes. In addition, many of the included studies were published before the introduction of the CONSORT guidelines for reporting RCTs, which may have influenced the quality of reporting.

## 8 Conclusion

The results of this systematic review suggest that cardiorespiratory and muscular strength exercise may have medium to large effects on symptoms of depression and anxiety among students in higher education facing mental health challenges. There is low-certainty evidence for the effect of exercise on anxiety sensitivity. Overall, these findings indicate a potential role for exercise as an intervention for students facing mental health challenges and suggest that it may be considered within student health services. However, the certainty of evidence was low to very low across all outcomes. High-quality RCTs with well-designed exercise interventions are warranted to more conclusively determine the effect of cardiorespiratory and muscle strength exercise on mental health in this population.
