# Supplementary material for: Effects of exercise on depression and anxiety in university students: a systematic review and meta-analysis
Source: Front Sports Act Living. 2026 May 29;8:1708741. doi: 10.3389/fspor.2026.1708741 (PMC13260482; doi:10.3389/fspor.2026.1708741)
Supplement: Supplementary file 1 [file Table1.docx]

**Appendix, Search strategy, 8. April 2025**

**Embase <1980 to 2025 Week 14>;Ovid MEDLINE(R) ALL <1946 to April 04, 2025>
APA PsycInfo <1806 to March 2025 Week 5> - via Ovid:**

/ = Exact Subject Headings, exp includes any narrowing headings

| **Concepts, elements used in the search strategy, including limitations** | **#** | **Query** | **Results from 8 Apr 2025** |
| --- | --- | --- | --- |
|  | 1 | (student*.ti. and (universit* or college* or "higher education" or graduate or undergraduate).ab.) or (Student* adj3 (universit* or college* or "higher education" or graduate or undergraduate)).ab. | 434,941 |
|  | 2 | exp Students, Health Occupations/ or graduate student/ or social work student/ or exp health student/ or non-medical student/ or undergraduate student/ or university student/ or veterinary student/ or college student/ or Business Students/ or exp College Students/ or Dental Students/ or Law Students/ or Medical Students/ or College Graduates/ or Graduate Students/ | 451,204 |
| Students | 3 | or/1-2 | 702,551 |
|  | 4 | exp exercise/ or exp physical activity/ | 1,286,739 |
|  | 5 | ("physical activity" or exercise or running or walking or swimming or cycling or bicycling or dance or dancing or jogging or "physical work−out" or "physical workout" or "fitness workout" or "fitness work-out" or kinesiotherap* or "fitness class*" or spinning).tw,kf,id. | 1,847,384 |
|  | 6 | ((aerobic or interval or strength or Endurance or Resistance or fitness) adj4 training).tw,kf,id. | 98,586 |
|  | 7 | exp kinesiotherapy/ or exp exercise therapy/ | 179,385 |
| PA | 8 | or/4-7 | 2,360,291 |
|  | 9 | ("mental health" or anxiety or depress* or stress or "quality of life" or wellbeing or well-being or mood or moods or affect*).hw,tw,kf,id. | 13,214,690 |
|  | 10 | Mental Disorders/ or mental disease/ | 596,242 |
|  | 11 | (psychological* adj4 (problem* or outcome* or challeng* or function* or symptom* or health or status)).tw,kf,id. | 249,535 |
|  | 12 | (mental* adj2 (disease* or ill* or disorder*)).tw,kf,id. | 422,775 |
|  | 13 | Emotional States/ use psyh or exp Affect/ | 189,646 |
| Mental health | 14 | or/9-13 | 13,657,140 |
|  | 15 | ("0200" or "0240" or "0280" or "0300" or "0400").pt. use psyh | 1,135,808 |
|  | 16 | (books or chapter or conference* or editorial).pt. use emez | 7,112,172 |
|  | 17 | letter.pt. use medall | 1,290,478 |
| Publication types | 18 | or/15-17 | 9,538,458 |
|  | 19 | (trial* or effect* or outcome* or efficacy).ti. | 8,137,235 |
|  | 20 | ((experimental* or non-randomi* or nonrandom* or controlled*) adj3 (study or trial*)).ti,ab. | 1,519,526 |
|  | 21 | intervention*.ti,ab. | 4,181,917 |
|  | 22 | exp randomized controlled trial/ or randomization/ or Random Allocation/ or Randomized Controlled Trial.pt. | 1,672,796 |
|  | 23 | (allocated adj2 random*).ab. or (randomi* or randomly).ti,ab. or "control* group*".ab. | 4,408,258 |
| Intervention studies | 24 | or/19-23 | 14,296,429 |
|  | 25 | (qualitative or phenomenolog* or interview* or experience* or "focus group*" or themes or thematic or "content analys*" or ethnograph* or "grounded theor*" or Hermeneutic*).tw,hw,id,kf. | 6,558,219 |
|  | 26 | ("1300" or "1600").md. use psyh | 369,855 |
| Qualitative studiers | 27 | or/25-26 | 6,622,507 |
| Intervention, or qualitative | 28 | 24 or 27 | 19,301,198 |
| Concepts combined | 29 | 3 and 8 and 14 and 28 | 7,246 |
| **Removing publication types** | **30** | **29 not 18** | **5,833** |
| ***Remove internal duplicates*** | ***31*** | ***remove duplicates from 30*** | ***3,962*** |

**Link, Access is needed**

<https://ovidsp.ovid.com/ovidweb.cgi?T=JS&NEWS=N&PAGE=main&SHAREDSEARCHID=6oQXXsIyoLLczUN4kNX38QOijcTuxKgIVBg1gIr6YfdZyW4zjOb4jy142KhxZJAJV>

**Results:**

| 30 | 29 not 18  [Embase <1980 to 2025 Week 14>](https://ovidsp.dc1.ovid.com/ovid-new-a/ovidweb.cgi?Titles+Display=G\|S.sh.178\|1&S=KNMHFPFEDDACLAKEKPIJAGMIMAGEAA00)  [Ovid MEDLINE(R) ALL <1946 to April 04, 2025>](https://ovidsp.dc1.ovid.com/ovid-new-a/ovidweb.cgi?Titles+Display=G\|S.sh.178\|2934&S=KNMHFPFEDDACLAKEKPIJAGMIMAGEAA00)  [APA PsycInfo <1806 to March 2025 Week 5>](https://ovidsp.dc1.ovid.com/ovid-new-a/ovidweb.cgi?Titles+Display=G\|S.sh.178\|4722&S=KNMHFPFEDDACLAKEKPIJAGMIMAGEAA00) | **5833**  2933  1788  1112 |
| --- | --- | --- |

**CINAHL (EBSCO*host*: Search modes - Find all my search terms). 8. April 2025**

The default fields for unqualified searches consist of the following: Title, Abstract and Subject headings, PubMed ID (PMID), Digital Object Identifier, Author. Boolean OR/or are the same. XB = words from title or abstract, word indexed. N2 = adj3 (Ovid). MH exact subject CINAHL headings, + includes any narrowing headings

|  | **#** | **Query** | **Limiters/Expanders** | **Results** |
| --- | --- | --- | --- | --- |
|  | S1 | TI (student*) AND AB (universit* or college* or "higher education" or graduate or undergraduate) |  | 34,900 |
|  | S2 | AB (Student* N2 (universit* or college* or "higher education" or graduate or undergraduate)) |  | 41,975 |
|  | S3 | (MH "Students, Undergraduate") OR (MH "Students, Health Occupations+") OR (MH "Students, Graduate+") OR (MH "Students, College+") |  | 143,662 |
| **Student** | S4 | S1 OR S2 OR S3 |  | 162,123 |
|  | S5 | exercise OR MH "Exercise+" |  | 257,045 |
|  | S6 | "physical activity" or running or walking or swimming or cycling or bicycling or dance or dancing or jogging or "physical work−out" or "physical workout" or "fitness workout" or "fitness work-out" or "fitness class*" or spinning |  | 192,863 |
|  | S7 | (aerobic or interval or strength or Endurance or Resistance or fitness) N3 training |  | 23,525 |
|  | S8 | kinesiotherap* |  | 309 |
| **PA** | S9 | S5 OR S6 OR S7 OR S8 |  | 366,732 |
|  | S10 | MH "Mental Disorders+" |  | 685,640 |
|  | S11 | "mental health" or stress or "quality of life" or wellbeing or well-being or mood or moods or affect* |  | 1,098,458 |
|  | S12 | anxiety or depress* |  | 309,882 |
|  | S13 | psychological* N3 (problem* or outcome* or challeng* or function* or symptom* or health or status) |  | 34,896 |
|  | S14 | (mental* N1 (disease* or ill* or disorder*)) |  | 108,303 |
| **Mental health** | S15 | S10 OR S11 OR S12 OR S13 OR S14 |  | 1,645,263 |
|  | S16 | TI (trial* or effect* or outcome* or efficacy) OR MH ("Experimental Studies+") OR PT ("randomized controlled trial") |  | 1,117,255 |
|  | S17 | XB ((experimental* or non-randomi* or nonrandom* or controlled*) N2 (study or trial*)) |  | 243,742 |
|  | S18 | XB (intervention*) |  | 605,944 |
|  | S19 | AB (allocated N1 random*) OR XB (randomi* or randomly) OR AB ("control* group*") |  | 495,959 |
| Intervention studies | S20 | S16 OR S17 OR S18 OR S19 |  | 1,693,177 |
| Qualitative studies | S21 | qualitative or phenomenolog* or interview* or experience* or "focus group*" or themes or thematic or "content analys*" or ethnograph* or "grounded theor*" or Hermeneutic* |  | 1,002,061 |
| **Intervention studies, or Qualitative studies** | S22 | S20 OR S21 |  | 2,435,460 |
| **Concpets combined** | S23 | S4 AND S9 AND S15 AND S22 |  | 1,523 |
| Publication types | S24 | PT ("dissertation"or "doctoral dissertation" or "editorial" or "letter") |  | 617,111 |
| ***Removing publication types*** | ***S25*** | ***S23 NOT S24*** |  | ***1,493*** |
| ***Exclude MEDLINE records*** | ***S26*** | ***S23 NOT S24*** | ***Limiters - Exclude MEDLINE records*** | ***1,096*** |

**SPORTDiscus (EBSCO*host*). Search modes - Find all my search terms. 8. April 2025**

Unqualified searches :Words from the database standard fields, includes words from title, abstract, subject, Word index. DE = Heading or Keyword. [Phrase Indexed]. Performs an exact search of the headings and subheadings listed in the records. SU =Subject Terms. [Word Indexed]. Performs a keyword search of the subject headings listed in the records.

| **#** | **Query** | **Results** |
| --- | --- | --- |
| S1 | TI (student*) AND AB (universit* or college* or "higher education" or graduate or undergraduate) | 12,971 |
| S2 | AB (Student* N2 (universit* or college* or "higher education" or graduate or undergraduate)) | 20,271 |
| S3 | DE ("COLLEGE students" OR "MEDICAL students" OR UNDERGRADUATES OR "WOMEN college students") OR SU ("COLLEGE students" OR "MEDICAL students" OR UNDERGRADUATES OR "WOMEN college students") | 15,152 |
| S4 | S1 OR S2 OR S3 | 29,678 |
| S5 | exercise | 315,956 |
| S6 | running or walking or swimming or cycling or bicycling or dance or dancing or jogging or "physical work−out" or "physical workout" or "fitness workout" or "fitness work-out" or "fitness class*" or spinning | 315,085 |
| S7 | (aerobic or interval or strength or Endurance or Resistance or fitness) N3 training | 46,075 |
| S8 | kinesiotherap* | 8,137 |
| S9 | "physical activity" | 77,849 |
| S10 | S5 OR S6 OR S7 OR S8 OR S9 | 616,389 |
| S11 | "mental health" or stress or "quality of life" or wellbeing or well-being or mood or moods or affect* | 202,376 |
| S12 | anxiety or depress* | 39,834 |
| S13 | psychological* N3 (problem* or outcome* or challeng* or function* or symptom* or health or status) | 6,041 |
| S14 | mental* N1 (disease* or ill* or disorder*) | 8,169 |
| S15 | S11 OR S12 OR S13 OR S14 | 226,355 |
| S16 | TI (trial* or effect* or outcome* or efficacy) | 147,822 |
| S17 | XB ((experimental* or non-randomi* or nonrandom* or controlled*) N2 (study or trial*)) | 35,299 |
| S18 | XB (intervention*) | 87,390 |
| S19 | AB (allocated N1 random*) or XB (randomi* or randomly) or AB ("control* group*") | 81,702 |
| S20 | S16 OR S17 OR S18 OR S19 | 251,702 |
| S21 | qualitative or phenomenolog* or interview* or experience* or "focus group*" or themes or thematic or "content analys*" or ethnograph* or "grounded theor*" or Hermeneutic* | 217,371 |
| S22 | S20 OR S21 | 439,884 |
| S23 | S4 AND S10 AND S15 AND S22 | 1,119 |

**Scopus. 8. April 2025**

Search string, Advanced search:

((TITLE(student*) AND ABS(universit* or college* or "higher education" or graduate or undergraduate)) OR ABS(Student* w/2 (universit* or college* or "higher education" or graduate or undergraduate))) AND (TITLE-ABS-KEY("physical activity" or exercise or running or walking or swimming or cycling or bicycling or dance or dancing or jogging or "physical work−out" or "physical workout" or "fitness workout" or "fitness work-out" or kinesiotherap* or "fitness class*" or spinning) OR TITLE-ABS-KEY((aerobic or interval or strength or Endurance or Resistance or fitness) w/3 training)) AND (TITLE-ABS-KEY("mental health" or anxiety or depress* or stress or "quality of life" or wellbeing or well-being or mood or moods or affect*) OR TITLE-ABS-KEY(psychological* w/3 (problem* or outcome* or challeng* or function* or symptom* or health or status)) OR TITLE-ABS-KEY(mental* w/1 (disease* or ill* or disorder*))) AND (TITLE(trial* or effect* or outcome* or efficacy) OR TITLE-ABS((experimental* or non-randomi* or nonrandom* or controlled*) w/2 (study or trial*)) OR TITLE-ABS(intervention*) OR ABS("control* group*") OR KEY(randomized) OR ABS(allocated w/2 random*) OR TITLE-ABS(randomi* or randomly) OR TITLE-ABS-KEY(qualitative or phenomenolog* or interview* or experience* or "focus group*" or themes or thematic or "content analys*" or ethnograph* or "grounded theor*" or Hermeneutic*))

**Result: 3514 records, 8. April 2025**
