# Supplementary material for: Effects of exercise on depression and anxiety in university students: a systematic review and meta-analysis
Source: Front Sports Act Living. 2026 May 29;8:1708741. doi: 10.3389/fspor.2026.1708741 (PMC13260482; doi:10.3389/fspor.2026.1708741)
Supplement: Supplementary file 2 [file Table2.docx]

**Supplementary file 2**

| **Overview of excluded studies** |  |
| --- | --- |
| **Studies identified via databases** | |
|  | Reason for exclusion |
| 1. Wen, A. (2007). Changes of depressive symptom and cardiorespiratory function in undergraduate students with severe depression following the intervention with short-term aerobic exercise prescription. *Journal of Clinical Rehabilitative Tissue Engineering Research*, *11*(17), 3316-3318 | Not published in English |
| 1. Yamazaki, F., Yamada, H. & Morikawa, S. (2013). [Influence of an 8-week exercise intervention on body composition, physical fitness, and mental health in female nursing students]. *Journal of UOEH*, *35*(1), 51-58 | Not published in English |
| 1. Yin, X. W., Yu, X. X., Yin, G. C. & Qiu, D. M. (2006). Effects of sport-cognition intervention on the mental health of college students. *Chinese Journal of Clinical Rehabilitation*, *10*(38), 39-41 | Not published in English |
| 1. Guan, S.-y., Li, Q.-l., & Zhang, S.-s. (2008). Effect of strength training on depression and manoamine transmitters in female college students with depression symptoms. *Journal of Wuhan Institute of Physical Education*, 42(10), 70-74. | Not published in English |
| 1. Chunlei, Z., Shouwei, Z., & Kunpeng, X. (2016). Influence of Exercise Intervention on College Students' Psychological Pressure: Mediating Effect on Health Belief. *Journal of Chengdu Sport University*, 42(4), 103-108. | Not published in English |
| 1. Cai, Y., Ning, L., Wang, G., Lei, Y., & Long, L. (2019). Effect of exercise prescription and mobile APP for health education on the physical self-esteem and mental health among female college students. *Chinese Journal of School Health*, 40(10), 1503-1505. | Not published in English |
| 1. Hu, Q. (2019). The Effect of increased intensity of physical exercises on mental health and resilience among college students. *Chinese Journal of School Health*, 40(1), 83-85. | Not published in English |
| 1. Ren, P. H., & Nie, M. (2023). Intervention effects with esports and exercise on body mass index and sleep quality of depressed college students. *Chinese Journal of School Health*, 44(8), 1190-1193. | Not published in English |
| 1. Tang, W.-j., & Zhang, W. (2008). A Functional Analysis of Physical Exercises Intervention in Depressive Mood of College Students. *Journal of Beijing Sport University*, 31(11), 1534-1536. | Not published in English |
| 1. 沈翔. (2015). Effects of Exercise Intervention and Exercise-Cognition Intervention on Stress Coping and Psychological Capability of College Students., 49(10), 76-82. | Not published in English |
| 1. Hoyos-Cifuentes, J. D., & Bernal-Torres, C. A. (2021). Analysis of physical activity benefits in crisis situations for university students with depressive symptoms. *Formacion Universitaria*, 14(6), 175-182. | Not published in English |
| 1. Cao, G. M., & Cao, H. (2002). An experimental study on the relationship between physical exercises and the college students' mental health. *Journal of Wuhan Institute of Physical Education*, 36(1), 131;146-131;146. | Not published in English |
| 1. Yifan, Z., Yuiing, G., Donghui, T., & Yuan, G. (2018). Effects of Acute Aerobic Exercise on Emotion Regulation Ability in Female College Students with Anxiety: Potential Mechanism and Influencing Factors. *Journal of Tianjin University of Sport / Tianjin Tiyu Xueyuan Xuebao*, 33(3), 210-216. | Not published in English |
| 1. Antony, V. C. & Tomar, R. (2019). Assessing the effect of fartlek training on perceived wellness of university students of Saudi Arabia: A randomized controlled trial. *International Journal of Pharmaceutical Sciences and Research*, *10*(11), 5051-5056 | Wrong population |
| 1. Gao, F. (2020). Multidimensional effects of exercise intervention on mental health of college students. *Revista Argentina de Clinica Psicologica*, 29(2), 1109-1116. | Wrong population |
| 1. Ghorbani, F., Heidarimoghadam, R., Karami, M., Fathi, K., Minasian, V. & Bahram, M. E. (2014). The effect of six-week aerobic training program on cardiovascular fit-ness, body composition and mental health among female students. *Journal of Research in Health Sciences*, 14(4), 264-267. | Wrong population |
| 1. Herbert, C., Meixner, F., Wiebking, C. & Gilg, V. (2020). Regular physical activity, short-term exercise, mental health, and well-being among university students: The results of an online and a laboratory study. *Frontiers in Psychology*, 11. | Wrong population |
| 1. Ince, M. L. (2008). Use of a social cognitive theory-based physical-activity intervention on health-promoting behaviors of university students. Perceptual and Motor Skills, 107(3), 833-836. | Wrong population |
| 1. Johnston, S. A., Roskowski, C., He, Z., Kong, L. & Chen, W. (2021). Effects of team sports on anxiety, depression, perceived stress, and sleep quality in college students. Journal of American college health : J of ACH, 69(7), 791-797. | Wrong population |
| 1. Kim, I. & Ahn, J. (2021). The effect of changes in physical self-concept through participation in exercise on changes in self-esteem and mental well-being. International Journal of Environmental Research and Public Health, 18(10), 5224. | Wrong population |
| 1. Ma, J., Williams, J., Morris, P. G. & Chan, P. S. W. Y. (2022). Effectiveness of mindful walking intervention in nature on sleep quality and mood among university student during Covid-19: A randomised control study. Explore. | Wrong population |
| 1. Lee, K., Bae, H. & Jang, S. (2022). Effect of Exercise Combined with Natural Stimulation on Korean College Students' Concentration and Positive Psychological Capital: A Pilot Study. Healthcare (Basel, Switzerland), 10(4). | Wrong population |
| 1. McEntee, D. J. & Halgin, R. P. (1999). Cognitive group therapy and aerobic exercise in the treatment of anxiety. Journal of College Student Psychotherapy, 13(3), 37-55. | Wrong population |
| 1. Meier, N. F. & Welch, A. S. (2016). Walking versus biofeedback: a comparison of acute interventions for stressed students. Anxiety, stress, and coping, 29(5), 463-478. | Wrong population |
| 1. Morris, A., Do, D., Gottlieb-Smith, R., Ng, J., Jain, A., Wright, S. & Shochet, R. (2012). Impact of a fitness intervention on medical students. Southern Medical Journal, 105(12), 630-63. | Wrong population |
| 1. Murray, A., Marenus, M., Cahuas, A., Friedman, K., Ottensoser, H., Kumaravel, V., Sanowski, J. & Chen, W. (2022). The Impact of Web-Based Physical Activity Interventions on Depression and Anxiety Among College Students: Randomized Experimental Trial. JMIR formative research, 6(4), e31839. | Wrong population |
| 1. O'Brien, N., Lawlor, M., Chambers, F. & O'Brien, W. (2020). State of mind ireland-higher education: A mixed-methods longitudinal evaluation of a positive mental health intervention. International Journal of Environmental Research and Public Health, 17(15), 1-23. | Wrong population |
| 1. Rosales-Ricardo, Y., Caiza-Ruiz, M. V., Sanchez-Canizares, M. & Ferreira, J. P. (2021). Physical exercise on Burnout Syndrome levels and Heart Rate Variability in university students: an intervention study. Medicina (Brazil), 54(4). | Wrong population |
| 1. Shaposhnikova, I. I., Korsun, S. M., Arefieva, L. P., Kostikova, O. V., Serhiienko, V. M., Korol, S. A. & Riabchenko, V. G. (2021). Analysis of students somatic health and emotional state during sport games classes. Wiadomosci lekarskie 74(3), 608-612. | Wrong population |
| 1. Sharp, P. & Caperchione, C. (2016). The effects of a pedometer-based intervention on first-year university students: A randomized control trial. Journal of American college health : J of ACH, 64(8), 630-638. | Wrong population |
| 1. Stein, P. N. & Motta, R. W. (1992). Effects of aerobic and nonaerobic exercise on depression and self-concept. Perceptual and Motor Skills, 74(1), 79-89. | Wrong population |
| 1. Tucker, L. A. & Maxwell, K. (1992). Effects of weight training on the emotional well-being and body image of females: Predictors of greatest benefit. American Journal of Health Promotion, 6(5), 338-371 | Wrong population |
| 1. Waechter, R., Stahl, G., Rabie, S., Colak, B., Johnson-Rais, D., Landon, B., Petersen, K., Davari, S., Zaw, T., Mandalaneni, K. & Punch, B. (2021). Mitigating medical student stress and anxiety: Should schools mandate participation in wellness intervention programs? Medical teacher, 43(8), 945-955. | Wrong population |
| 1. Wang, J. & Li, C. (2022). Research on the Influence of Physical Exercise Health on the Mental Health of University Students. Journal of Environmental and Public Health, 2022, 6259631. | Wrong population |
| 1. Welford, P. & O'Brien, A. (2019). Improving student mental wellbeing. The BMJ, 366, l2421. | Wrong population |
| 1. Williams, P. A. & Cash, T. F. (2001). Effects of a circuit weight training program on the body images of college students. International Journal of Eating Disorders, 30(1), 75-82. | Wrong population |
| 1. Worobetz, A., A, O. R., Casey, M., Hayes, P., M, O. C., Walsh, J. C., Bengoechea, E. G., Woods, C., McGrath, D. & Glynn, L. G. (2022). Lessons learned from a pandemic: implications for a combined exercise and educational programme for medical students. BMC medical education, 22(1), 255. | Wrong population |
| 1. Worobetz, A., O'Callaghan, M., Walsh, J., Casey, M., Hayes, P., Bengoechea, E. G., Woods, C., McGrath, D. & Glynn, L. G. (2022). Exercise Compared to Mindfulness for Physical and Mental Wellbeing in Medical Students. Irish medical journal, 115(3), 560. | Wrong population |
| 1. Worobetz, A., Retief, P. J., Loughran, S., Walsh, J., Casey, M., Hayes, P., Bengoechea, E. G., O'Regan, A., Woods, C., Kelly, D., Connor, R. O., Grath, D. M. & Glynn, L. G. (2020). A feasibility study of an exercise intervention to educate and promote health and well-being among medical students: the 'MED-WELL' programme. BMC medical education, 20(1), 183. | Wrong population |
| 1. Yalman, A., Telli Atalay, O., Unver, F., Senol, H. & Taskin, H. (2021). The sub-acute effects of high-intensity interval training in healthy young adults: respiratory parameters, aerobic capacity and perceived stress. The Journal of sports medicine and physical fitness, 61(5), 617-624. | Wrong population |
| 1. Yang, P., Yang, H., Cao, Y., Yang, X. & He, B. (2022). Psychophysiological effects of rhythmic music combined with aerobic exercise in college students with minimal depressive symptoms. Sports Medicine and Health Science. | Wrong population |
| 1. Yorks, D. M., Frothingham, C. A. & Schuenke, M. D. (2017). Effects of Group Fitness Classes on Stress and Quality of Life of Medical Students. The Journal of the American Osteopathic Association, 117(11), e17-e25. | Wrong population |
| 1. Zhang, H. & Luo, S. (2020). Promoting effects of water sports on the mental health of college students. Revista Argentina de Clinica Psicologica, 29(2), 577-583. | Wrong population |
| 1. Zhang, H. & Luo, S. (2020a). Empirical analysis of the influence of swimming training on mental health and positive emotions of college students. Revista Argentina de Clinica Psicologica, 29(1), 242-248. | Wrong population |
| 1. Zhao, C. & Wang, Z. (2020). Influence of physical exercise on mental health and positive emotions of college students. Revista Argentina de Clinica Psicologica, 29(1), 1234-1239. | Wrong population |
| 1. Annesi, J. J., Porter, K. J., Hill, G. M., & Goldfine, B. D. (2017). Effects of Instructional Physical Activity Courses on Overall Physical Activity and Mood in University Students. Research quarterly for exercise and sport, 88(3), 358-364. | Wrong population |
| 1. Asci, F. H. (2003). The effects of physical fitness training on trait anxiety and physical self-concept of female university students. Psychology of Sport and Exercise, 4(3), 255-264. | Wrong population |
| 1. Barrantes-Brais, K., & Balaguer-Sola, I. (2019). Effect of positive psychology and exercise interventions on college students' psychological well-being and ill-being. Efecto de intervenciones de psicologia positiva y ejercicio fisico en el bienestar y malestar psicologico de estudiantes universitarios., 38(2), 149-177. | Wrong population |
| 1. Berger, B. G., & Owen, D. R. (1992). Preliminary analysis of a causal relationship between swimming and stress reduction: Intense exercise may negate the effects. International Journal of Sport Psychology, 23(1), 70-85. | Wrong population |
| 1. Faro, J., Wright, J. A., Hayman, L. L., Hastie, M., Gona, P. N., & Whiteley, J. A. (2019). Functional Resistance Training and Affective Response in Female College-Age Students. Medicine and science in sports and exercise, 51(6), 1186-1194. | Wrong population |
| 1. Friedman, K., Marenus, M. W., Murray, A., Cahuas, A., Ottensoser, H., Sanowski, J., & Chen, W. (2022). Enhancing Physical Activity and Psychological Well-Being in College Students during COVID-19 through WeActive and WeMindful Interventions. International Journal of Environmental Research and Public Health, 19(7), 4144. | Wrong population |
| 1. Huang, G. (2021). The effect of aerobic exercise on the mental health of college students. Revista de Psicologia del Deporte, 30(4), 132-139. | Wrong population |
| 1. Liu, C. (2022). Research on the influence of college students' participation in sports activities on their sense of inferiority based on self-esteem and general self-efficacy. Frontiers in Psychology, 13, 994209. | Wrong population |
| 1. Liu, M., & Shi, B. (2023). The effect of physical exercise on the anxiety of college students in the post-pandemic era: The mediating role of social support and proactive personality. Frontiers in Psychology, 14, 1128748 | Wrong population |
| 1. O'Connor, P. J., Petruzzello, S. J., Kubitz, K. A., & Robinson, T. L. (1995). Anxiety responses to maximal exercise testing. British Journal of Sports Medicine, 29(2), 97-102. | Wrong population |
| 1. Wang, J., & Li, C. (2022). Research on the Influence of Physical Exercise Health on the Mental Health of University Students. Journal of Environmental and Public Health, 2022, 6259631 | Wrong population |
| 1. Ren, H., & Song, Y.-e. (2020). Effects of physical exercises in varied intensities on mental health of college students. Revista Argentina de Clinica Psicologica, 29(2), 1074-1083. | Wrong population |
| 1. Wei, M. X., & Liu, Z. (2023). A Questionnaire-Based Study to Compare the Psychological Effects of 6 Weeks of Exercise in 123 Chinese College Students. Medical science monitor basic research, 29, e939096. | Wrong population |
| 1. Willeboordse, S. (2023). Effects of basketball and Baduanjin exercise interventions on problematic smartphone use and mental health among college students: a randomized controlled trial. Chinesische Medizin, 38(1), 26-29. | Wrong population |
| 1. Xiao, T., Jiao, C., Yao, J., Yang, L., Zhang, Y., Liu, S., Grabovac, I., Yu, Q., Kong, Z., Yu, J. J., & Zhang, J. (2021). Effects of Basketball and Baduanjin Exercise Interventions on Problematic Smartphone Use and Mental Health among College Students: A Randomized Controlled Trial. Evidence-based Complementary and Alternative Medicine, 2021, 8880716. | Wrong population |
| 1. Zhang, T., Liu, H., Lu, Y., & Wang, Q. (2023). The Nexus of Sports-Based Development and Education of Mental Health and Physical Fitness. International Journal of Environmental Research and Public Health, 20(4), 3737 | Wrong population |
| 1. Berger, B. G., & Friedman, E. (1988). Comparison of Jogging, the Relaxation Response, and Group Interaction for Stress Reduction. Journal of sport & exercise psychology, 10(4), 431-447. | Wrong population |
| 1. Calazans de Lira, C. T., Santiago, L. d. C. S., Henrique, R. d. S., Rangel-Junior, J. F. L. B., Campello, C. P., & Santos, M. A. M. (2024). Examining the Effects of Strength Training with Load Progression on Sleep Parameters and Mental Health in College Students. *Sleep science* (Sao Paulo, Brazil), 17(2), e134-e142. | Wrong population |
| 1. Chaoke Y. Optimal Exercise Strategies for Enhancing University Students' Mental Health. Revista Internacional de Medicina y Ciencias de la Actividad Fisica y del Deporte (2025) 25(100):231-44. | Wrong population |
| 1. Hsieh, P. L. (2011). A school-based health promotion program for stressed nursing students in Taiwan. The journal of nursing research : JNR, 19(3), 230-237. | Wrong outcome measure |
| 1. Hurdiel, R., Watier, T., Honn, K., Peze, T., Zunquin, G. & Theunynck, D. (2017). Effects of a 12-week physical activities programme on sleep in female university students. Research in sports medicine (Print), 25(2), 191-196. | Wrong outcome measure |
| 1. Jawwad, G., Khan, H. F., Iftikhar, M., Hussain, A., Arshad, S. & Siddique, L. (2022). Exercise Induces Autonomic and Neuro-endocrine Response among Psychologically Stressed Medical Students. Pakistan Journal of Medical and Health Sciences, 16(6), 135-137. | Wrong outcome measure |
| 1. Rosales-Ricardo, Y. & Ferreira, J. P. (2022). Effects of Physical Exercise on Burnout Syndrome in University Students. MEDICC Review, 24(1), 36-39. | Wrong outcome measure |
| 1. Margulis, A., Andrews, K., He, Z. & Chen, W. (2021). The effects of different types of physical activities on stress and anxiety in college students. Current Psychology: A Journal for Diverse Perspectives on Diverse Psychological Issues, No-Specified. | Wrong study design |
| 1. deJonge, M. L., Jain, S., Faulkner, G. E., & Sabiston, C. M. (2021). On campus physical activity programming for post-secondary student mental health: Examining effectiveness and acceptability. Mental Health and Physical Activity, 20, 100391. | Wrong study design |
| 1. Luo, X. (2020). Intervention effect of long-distance running on depression of colleges students. Revista Argentina de Clinica Psicologica, 29(2), 90-95. | Wrong study design |
| 1. McFadden, T., Fortier, M. S., & Guerin, E. (2017). Investigating the effects of Physical Activity Counselling on depressive symptoms and physical activity in female undergraduate students with depression: A multiple baseline single-subject design. Mental Health and Physical Activity, 12, 25-36. | Wrong study design |
| 1. Yates, B. E., DeLetter, M. C., & Parrish, E. M. (2020). Prescribed exercise for the treatment of depression in a college population: An interprofessional approach. Perspectives in psychiatric care, 56(4), 894-899. | Wrong study design |
| 1. Zuo, K., & Yue, Q. (2020). Improvement of mental health and depression of college students by physical exercise. Revista Argentina de Clinica Psicologica, 29(1), 373-378. | Wrong study design |
| 1. Wang, L., Li, J., Bai, S., Liu, T., Pei, T., Liu, Z., Wang, L., Yang, D., Ruan, C., Trincone, A., & Gong, M. (2019). The effect of different exercise on anxiety and depression of college students (Vol. 2079). *American Institute of Physics Inc.* | Wrong study design |
| 1. Ma G. Physical Exercise Intervention on Mental Health and Negative Emotions of College Students. Revista Argentina de Clinica Psicologica (2020) 29(1):1039-44. | Wrong study design |
| 1. Korman, N., Fox, H., Skinner, T., Dodd, C., Suetani, S., Chapman, J., Parker, S., Dark, F., Collins, C., Rosenbaum, S. & Siskind, D. (2020). Feasibility and Acceptability of a Student-Led Lifestyle (Diet and Exercise) Intervention Within a Residential Rehabilitation Setting for People With Severe Mental Illness, GO HEART (Group Occupation, Health, Exercise And Rehabilitation Treatment). *Frontiers in Psychiatry*, 11, 319. | Wrong intervention |
| 1. Yu, L., Xia, J. M., Zhang, L., Chu, W., & Defilla, S. (2021). Effect of prescription of traditional sports regimen on depression of college students (Vol. 233). EDP Sciences. | Wrong intervention |
| 1. Mailey EL, Wojcicki TR, Motl RW, Hu L, Strauser DR, Collins KD, et al. Internet-Delivered Physical Activity Intervention for College Students with Mental Health Disorders: A Randomized Pilot Trial. *Psychology, health & medicine* (2010) 15(6):646-59. | Wrong intervention |
| 1. Keeler LA, Skidmore B, Leenstra T, MacDonald JR, Stewart D. Treating University Students' Depression Using Physical Activity with Peers: Two Field-Based Quasi-Experiments Grounded in the Self-Determination Theory. *Journal of College Student Psychotherapy* (2021) 35(3):205-23. | Wrong intervention |
| 1. Berger, B. G. (1987). Exercice physique et reduction du stress. Physical exercise and stress reduction.(3), 25-29. | Not an original paper |
| 1. Harris, D. V., Rueter, M., & Mutrie, N. (1985). The effects of running on individuals who are clinically depressed. | Not an original paper |
| **Studies identified from citation searching** | |
| 1. Ma MK. Evaluation of the effect of different exercise programs on the intervention of zhuang depression and anxiety college students. Chin J Health 2017;38:112–4. | Chinese language |
| 1. Zhao HQ. Effects of high-intensity intermittent exercise on depression in female college students. J Liao Cheng Univers (Nat Sci) 2017;30:88–92 | Chinese language |
| 1. Zhu l, Yu SB. Experimental study on the effect of middle-distance running exercise on depression symptoms of college students. J Huangshan Univers 2011;13:79–82. | Chinese language |
| 1. Liu, Y.; LI, X.; WANG, X.; YANG, N.; WAN, B.; SHI, B. Mediating Effect of Exercise Intervention on Self-Efficacy of Negative Emotion Regulation of Home Schooled Students during the COVID-19 Pandemic. J. Beijing Sport Univ. 2020, 43, 76–83. | Chinese language |
| 1. Anshel, M. H. (1996). Effect of Chronic Aerobic Exercise and Progressive Relaxation on Motor Performance and affect following Acute Stress. Behav Med, 21(4), 186-196. | Wrong study population |
| 1. Bargi, G. (2022). Effectiveness of Physical Activity Counseling in University Students Educated by Distance Learning During COVID-19 Pandemic: A Randomized-Controlled Trial. Journal of Basic and Clinical Health Sciences, 6(2), 374-384. | Wrong study population |
| 1. Blough, J., & Loprinzi, P. D. (2018). Experimentally investigating the joint effects of physical activity and sedentary behavior on depression and anxiety: A randomized controlled trial. J Affect Disord, 239, 258-268. | Wrong study population |
| 1. Herring, M. P., Jacob, M. L., Suveg, C., Dishman, R. K., & O’Connor, P. J. (2012). Feasibility of Exercise Training for the Short-Term Treatment of Generalized Anxiety Disorder: A Randomized Controlled Trial. Psychother Psychosom, 81(1), 21-28. | Wrong study population |
| 1. Hopkins, M. E., Davis, F. C., Vantieghem, M. R., Whalen, P. J., & Bucci, D. J. (2012). Differential effects of acute and regular physical exercise on cognition and affect. Neuroscience, 215, 59-68. | Wrong study population |
| 1. Mohammadi, M., & Abhar. (2011). A study and comparison of the effect of team sports (soccer and volleyball) and individual sports (table tennis and badminton) on depression among high school students. Australian journal of basic and applied sciences, 5, 1005-1011. | Wrong study population |
| 1. Broman-Fulks, J. J., Berman, M. E., Rabian, B. A., & Webster, M. J. (2004). Effects of aerobic exercise on anxiety sensitivity. Behav Res Ther, 42(2), 125-136. | Lack of control group |
| 1. Hamed, N. S., Abdel-aziem, A. A., Muhsen, B. A., Eid, M. M., Allam, H. A., El-Gendy, A. M., & El Sayyad, L. K. (2021). The effect of aerobic training versus cognitive behavioral therapy in management of anxiety, depression and stress related to COVID 19 pandemic among university students: A randomized controlled trial. Medical Science, 25(115), 2233-2246 | Lack of control group |
| 1. Chawla, G., Azharuddin, M., Ahmad, I., & Hussain, M. E. (2022). Effect of whole-body vibration on depression, anxiety, stress, and quality of life in college students: A randomized controlled trial. Oman medical journal, 37(4), 1-9. | Wrong intervention |
