# Supplementary material for: Effects of exercise on depression and anxiety in university students: a systematic review and meta-analysis
Source: Front Sports Act Living. 2026 May 29;8:1708741. doi: 10.3389/fspor.2026.1708741 (PMC13260482; doi:10.3389/fspor.2026.1708741)
Supplement: Supplementary file 3 [file Table3.docx]

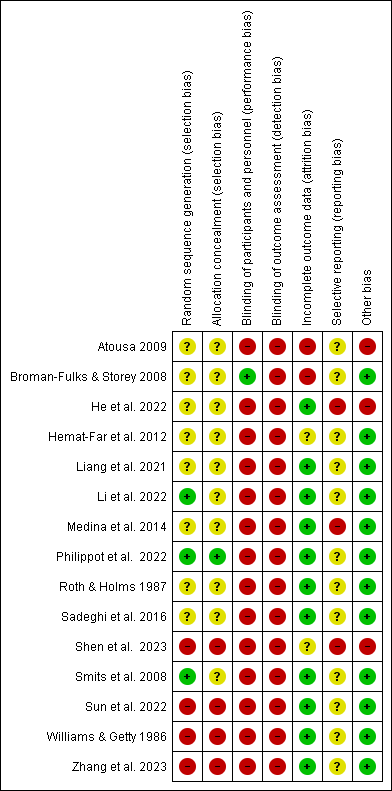


**Supplementary file 3** Risk of bias summary: the review authors judgment of the risk of bias of each included study
